# Clinical Significance of Molecular Alterations and Systemic Therapy for Meningiomas: Where Do We Stand?

**DOI:** 10.3390/cancers14092256

**Published:** 2022-04-30

**Authors:** Alessia Pellerino, Francesco Bruno, Rosa Palmiero, Edoardo Pronello, Luca Bertero, Riccardo Soffietti, Roberta Rudà

**Affiliations:** 1 Division of Neuro-Oncology, Department Neuroscience, University and City of Health and Science Hospital, 10126 Turin, Italy; alessia.pellerino85@gmail.com (A.P.); f.bruno@unito.it (F.B.); rosapalmiero61@gmail.com (R.P.); rudarob@hotmail.com (R.R.); 2 Department of Neurology Unit, Department of Translational Medicine, University of Eastern Piedmont, 28100 Novara, Italy; pronello.edoardo@gmail.com; 3 Pathology Unit, Department of Medical Sciences, University and City of Health and Science Hospital, 10126 Turin, Italy; luca.bertero@unito.it; 4 Department of Neurology, Castelfranco Veneto and Treviso Hospital, 31100 Treviso, Italy

**Keywords:** chemotherapy, immunotherapy, recurrent meningioma, *AKT*, *PIK3CA*, *SMO*

## Abstract

**Simple Summary:**

Meningiomas are the most frequent intracranial tumors and comprise a heterogeneous spectrum of diseases, ranging from small, asymptomatic tumors that do not need treatment to large, symptomatic ones causing seizures or neurological deficits that require surgery and/or radiotherapy. Systemic therapy is reserved for progressive or recurrent meningiomas when surgery and/or radiotherapy options have been exhausted, with only modest activity in terms of disease control and survival. Novel molecular alterations are correlated with grading, location, and prognosis of meningiomas. Moreover, some of these driver alterations regulate meningioma growth and progression and may be targeted by specific drugs that are under investigation in clinical trials. Lastly, the microenvironment surrounding meningiomas may also contribute to regulating tumor growth: in particular, PD-L1 and/or M2 macrophage expression may represent a target for immunotherapy.

**Abstract:**

Meningiomas are common intracranial tumors that can be treated successfully in most cases with surgical resection and/or adjuvant radiotherapy. However, approximately 20% of patients show an aggressive clinical course with tumor recurrence or progressive disease, resulting in significant morbidity and increased mortality. Despite several studies that have investigated different cytotoxic agents in aggressive meningiomas in the past several years, limited evidence of efficacy and clinical benefit has been reported thus far. Novel molecular alterations have been linked to a particular clinicopathological phenotype and have been correlated with grading, location, and prognosis of meningiomas. In this regard, *SMO*, *AKT*, and *PIK3CA* mutations are typical of anterior skull base meningiomas, whereas *KLF4* mutations are specific for secretory histology, and *BAP1* alterations are common in progressive rhabdoid meningiomas. Alterations in *TERT*, *DMD*, and *BAP1* correlate with poor outcomes. Moreover, some actionable mutations, including *SMO*, *AKT1*, and *PIK3CA*, regulate meningioma growth and are under investigation in clinical trials. PD-L1 and/or M2 macrophage expression in the microenvironment provides evidence for the investigation of immunotherapy in progressive meningiomas.

## 1. Introduction

Meningiomas are the most frequent intracranial tumors, with an annual age-adjusted incidence rate of 9.12 per 100,000 population in 2014–2018 according to the CBTRUS report of 2021 [1]. The incidence of meningiomas increases with age, with a major prevalence after the age of 66 years. Meningiomas comprise a heterogenous spectrum of diseases with significant variability in tumor biology and clinical outcome, ranging from small and asymptomatic incidental meningiomas that are observed and do not need treatment to large, symptomatic meningiomas causing seizures or neurological deficits that require surgery and/or radiotherapy [2]. Most meningiomas are grade 1 tumors (94.6%), where the gross total resection is curative, with excellent long-term control rates; however, some clinical series with follow-up ranging from 5 to 10 years suggest the risk of underreporting late meningioma recurrences that can occur decades after primary treatment [3]. Grades 2 and 3 meningiomas represent 4.2% and 1.2%, respectively [4], with early and multiple relapses that require multimodality treatment, including repeated surgery and/or radiation therapy, and, in selected cases, chemotherapy or experimental clinical trials. In general, systemic therapy is reserved for grade 2 or 3 meningiomas as a last chance of treatment, when surgery and/or radiotherapy options have been exhausted, with only modest efficacy.

This review discusses the emerging role of genetic and epigenetic features as relevant biomarkers for outcome prediction, as well as advances in systemic therapies for intracranial meningiomas.

## 2. Molecular Features and Correlation with Histology and Grading

The WHO classification of 2021 identifies 15 different meningioma subtypes. Some diagnostic difficulties may occur when achieving a histological diagnosis of meningioma: for instance, different histological patterns can co-occur within the same tumor sample, posing challenges in terms of diagnostic interpretation and resulting in prognostic assessment [5]. Grade 1 meningiomas comprise nine variants: Meningothelial and fibroblastic variants are very common, while other variants, such as metaplastic- or lymphoplasmacyte-rich meningioma, are exceptionally rare. Grade 2 meningiomas include three histological subtypes, characterized by increased proliferation, nuclear pleomorphism, tumor necrosis, brain invasion, and an increased risk of recurrence. Grade 3 tumors comprise three histological subtypes—namely, papillary, rhabdoid, and anaplastic—and present a highly aggressive behavior and poor clinical course [6]. The histopathological criterion alone still leaves some uncertainty regarding the risk assessment in meningiomas [7]: It is now emphasized that the criteria defining atypical or anaplastic (i.e., grades 2 and 3) meningioma should be applied regardless of the underlying subtype. The inclusion of some novel molecular alterations in the diagnostic assessment may improve the identification of patients with a higher risk of recurrence who need close surveillance and/or more aggressive treatment.

Higher rates of copy-number alterations and karyotypic abnormalities are reported in anaplastic/malignant meningiomas, while fewer copy-number alterations are common in grade 1 meningiomas, although a small subset of these tumors harbors complex genomic rearrangements [8].

The most frequent alteration in meningioma is the loss of the neurofibromin 2 (*NF2*) gene on chromosome 22 [9,10]. The tumor suppressor gene *NF2* encodes the protein merlin (or schwannomin), which is correlated with the onset of schwannomas and meningiomas in the familial syndrome neurofibromatosis 2 [11] and is found in >50% of sporadic meningiomas [12,13,14]. The *NF2* inactivation is mostly due to LOH of chr22q and *NF2* mutation in other alleles, mitotic recombinations, or single or multiexon deletions, causing chromosomal instability that drives meningiomagenesis in a way that remains poorly understood [15]. Merlin negatively acts on multiple signaling pathways, including Hippo, Patched, and Notch pathways, and negatively regulates mammalian target of rapamycin complex 1 (*mTORC1*) [16] but positively influences the kinase activity of *mTORC2* [17].

A gene involved in the growth of *NF2*-negative meningiomas is *TRAF7*, an E3 ubiquitin ligase that interacts with MEKK3/MAP3K3 (mitogen-activated protein kinase kinase 3) and regulates apoptosis. *TRAF7* mutations were detected in up to 25% of grades 1 and 2 meningiomas [18,19]. A frequent aberration that can co-occur with *TRAF7* is *KLF4* mutations in up to 50% of *NF2*-nonmutated meningiomas of grade 1 [18,20,21]. Brastianos et al., using next-generation sequencing, showed that other genetic aberrations may be found in *NF2*-negative meningiomas, including mutations in *KDM5C*, *KDM6A*, and *SMARCB1* in 8% of patients, and the other six patients exhibited mutations of the PI3K–AKT–mTOR pathway, of whom five harbored *AKT1* mutations, and one, a novel *MTOR* mutation (p.Glu17Lys) [8]. Another study in 300 grades 1 and 2 meningiomas has found that 13% harbored the *AKT1 p.Glu17Lys* mutation [18] and displayed immunohistochemical evidence of PI3K–AKT–mTOR pathway activation. In addition, 1–5% of meningiomas without alterations in *NF2* and *AKT1*, harbor mutations in the *SMO* gene, which encodes smoothened homolog, a member of the Hedgehog signaling pathway [8,18,19]. SMO interacts with the suppressor of fused homolog (SUFU), causing the nuclear translocation of zinc-finger protein GLI1 (GLI1) and activation of target genes involved in cellular proliferation and angiogenesis [22]. Notably, the *PIK3CA*-mutant meningiomas lacking mutations in *NF2*, *AKT1*, and *SMO*, tend to express *TRAF7* mutations. Lastly, in the absence of any of the previously mentioned mutations, somatic mutations in *POLR2A* (encoding the DNA-directed RNA polymerase II subunit RPB1) may be found in about 6% of meningiomas. Typically, *POLR2A* is correlated with meningothelial histology, tumor location in the tuberculum sellae, and exclusive presence in grade 1 meningiomas [23]. In fact, the majority of genetic alterations listed above are found in grade 1 meningiomas only, while *NF2* mutations are the dominant molecular events (75%) in grade 2 meningiomas, followed by 9% harboring *TRAF7* or *PI3K* mutations, and 16% that do not contain any mutation [24].

Another important finding is the different role played by *TERT* mutations in favoring the progression of meningioma. In particular, the lack of *TERT* promoter mutations is the main characteristic of de novo grade 2 meningiomas, in contrast with the occurrence of *TERT* promoter mutations in secondary grade 2 meningiomas that have recurred from grade 1 [25]. NF2 alterations are the main finding also in grade 3 meningiomas. Vaubel et al. investigated the molecular features of rhabdoid meningiomas, which are designated as WHO grade 3 tumors, and found that the presence of a rhabdoid phenotype in the absence of other features of malignancy, such as high mitotic count and necroses, seems to define a clinical course comparable to grade 1 meningiomas [26]. Moreover, the identification of the inactivation of *BAP1* in rhabdoid meningiomas may differentiate between aggressive and less-aggressive rhabdoid-appearing meningiomas, where the loss of BAP1 protein expression indicates early tumor recurrence [27]. A genomic survey in a large, multi-institutional cohort of high-grade/progressive meningiomas has revealed at least three distinct patterns. The most common subtype was the *NF2*- mutated (NF2-associated pattern) which frequently harbored *CDKN2A/B* alterations and may be eligible for targeted therapies; in addition, the *NF2*-mutated pathway partly associated with *BAP1/PBRM1* alterations (rhabdoid/papillary histology) or skull-base disease (*NF2*-exclusive); lastly, the *NF2*-agnostic group harbored frequent *TERTp* and *TP53* mutations [28]. Recently, these genetic aberrations have been correlated with methylation classes in order to provide prognostic information not captured by previously established clinical and molecular factors [29,30,31]. In particular, Sahm et al. have shown that the classification of meningiomas based on DNA methylation profiling provides a more precise prediction of clinical behaviour than the WHO classification and grading system. Six methylation classes were identified, three of which were benign (MC ben-1, MC ben-2, MC ben-3), two were intermediate (MC int-A and MC int-B), and one was malignant (MC mal). Interestingly, WHO grade 1 meningiomas clustered in MC ben-1, MC ben-2, and MC ben-3 subgroups, while WHO grade 3 meningiomas fell into the MC mal subgroup, and WHO grade 2 meningiomas were scattered across all methylation classes [29]. Regarding histology, MC ben-1 contained mainly fibroblastic and psammomatous meningiomas, MC ben-2 was highly enriched for meningothelial meningiomas and almost all secretory meningiomas, and MC ben-3 included several subtypes but was particularly enriched for angiomatous meningiomas. Transitional meningiomas, and other rare entities, such as microcystic, chordoid, clear-cell, and metaplastic meningiomas, were distributed into several methylation classes. MC int-A and MC int-B mainly comprised atypical meningiomas, while anaplastic meningiomas predominantly fell into MC mal, and in a small proportion, in MC int-B or MC int-A. *NF2* inactivation alone was found in MC ben-1 and MC int A, or in association with *TERT* mutations in MC int-B and MC mal, while *TRAF7*, *KLF4*, *AKT1*, and *SMO* mutation were found in MC ben-2 only. MC ben-3 was not associated with any known mutation [29].

## 3. Molecular Features and Correlation with Location

The association between histological subtypes of meningiomas and their location are explained by embryonic reasons, as meninges at the skull base arise from the mesoderm, while meninges of the convexity derive from the neural crest. Therefore, meningothelial meningiomas preferentially develop from the skull base, while fibroblastic meningiomas arise primarily from convexity [32,33]. Grading is linked to the location of meningiomas, as grades 2 and 3 meningiomas are often located at the convexity or at parasagittal areas, whereas grade 1 meningiomas are mainly located at the skull base [34]. Meningiomas with *AKT1 p.Glu17Lys* mutations tend to have a skull-base or basal localization [35,36], *SMO*-mutated meningiomas predominate in the medial anterior skull base [18,37], *PIK3CA*-mutant meningiomas are preferentially localized at the skull base [19], and *POLR2A*-mutated meningiomas are mainly found in the tuberculum sellae region [23]. The presence of loss-of-function *SMARCE1* mutations is significantly associated with clear-cell histology in the spinal cord and cranial meningiomas [38,39]. Most intraventricular meningiomas (44%) harbor *NF2* mutations in the series of Jungwirth et al., while in non-*NF2*-mutated intraventricular meningiomas, genetic alterations including *TRAF7*, *AKT1*, *SMO*, *KLF4*, *PIK3CA*, and *TERT* are lacking, thus suggesting a role for alternative genes in the pathogenesis of non-NF2 intraventricular meningiomas. In fact, mutations of *APC*, *GABRA6*, *GSE1*, *KDR*, and two *SMO* missense mutations different from those previously reported have been found. Notably, all WHO grade 2 intraventricular meningiomas (n = 3) harbored *SMARCB1* and *SMARCA4* mutations [40]. An open question is whether the different embryological origin of meningiomas affects the sensitivity to drugs [41].

In a small proportion of patients, meningiomas arise as multiple and spatially distinct lesions and not as solitary tumors [42]. Multiple meningiomas may be associated with familial syndromes, such as neurofibromatosis type 2 (NF2) and familial meningiomatosis in patients with germline *NF2* and *SMARCB1* mutations [43]; however, Juratli et al. have reported a significantly lower frequency of *NF2* mutations in a series of 17 multiple meningiomas. All patients, with the exception of two cases, expressed *TRAF7*, *AKT1*, *SMO*, or *PIK3CA* mutations. In particular, the most frequent driver mutations were *TRAF7* (n  =  5); *PIK3CA*, *H1047R*, and *E545G* (n  =  3); *AKT1 E17K* (n  =  3); *NF2* (n  =  2); *SMO L412F* (n = 1); and *NF1* (n = 1), and one patient only did not harbor any driver mutation. Interestingly, the same mutation was not detected in different tumors from the same patient, suggesting genomically distinct molecular drivers and an independent origin of multiple meningiomas [44].

## 4. Molecular Features and Correlation with Prognosis

*TERT* promoter mutations have been correlated with shorter, progression-free survival in a retrospective series of 252 meningiomas, with 10.1 months in patients with *TERT* promoter mutations, compared with 179 months in patients without a *TERT* promoter mutations regardless of the histological grading [25]. Other studies have demonstrated the negative prognostic role of *TERT* mutations regardless of the WHO grading, suggesting that *TERT*-mutated meningiomas should be followed carefully, or treated aggressively, and include TERT analysis in the routine diagnostic assessment [45,46]. Furthermore, the loss of function of dystrophin-encoding and muscular dystrophy-associated gene (*DMD*) has been considered an additional negative prognostic factor in *TERT*-mutated meningiomas [47]. *SMO-* and *AKT-1*-mutated meningiomas have shown to recur more frequently, compared with meningiomas lacking *SMO* and *AKT1* mutations [37]. Furthermore, *AKT1 p.Glu17Lys* mutation confers a reduced time to tumor recurrence [20]. Conversely, a larger study on 3031 meningioma samples from 514 individual cases has shown that TRAF7, AKT1, and/or KLF4 mutations were significantly associated with a lower risk of progression [48].

DNA methylation analyses distinguish six clinically different meningioma groups—three with favorable outcomes, two with intermediate outcomes, and one with a poor outcome—representing a new approach for decisions regarding postoperative therapeutic interventions, in particular, whether to treat patients with adjuvant radiotherapy versus observation alone [29,30,31]. Recently, Berghoff et al. have investigated meningioma-relevant mutations and their correlation with DNA methylation clusters and patient survival. TRAKL pattern (any of the following mutations: *TRAF7*, *AKT1*, and *KLF4*) was predominantly found in methylation classes with favorable outcomes, while *NF2* was associated with methylation classes with poor outcomes. *TRAF7*, *KLF4*, and *TRAKL* mutation genotypes were associated with improved progression-free survival (PFS) and overall survival (OS), whereas *TERT* promoter methylation, and intermediate- and poor-outcome methylation classes, were associated with impaired PFS and OS. Methylation clustering showed better prognostic discrimination for PFS and OS than each of the individual mutations, where *TERT* mutation remained the unique independent significant prognostic factor for PFS in multivariable analysis [49]. Loss of *H3K27me3* has been reported as a prognostically unfavorable alteration in meningiomas: 13.9% (21/151) of meningiomas displayed the *H3K27me3* loss by immunohistochemistry (IHC) in a multicenter study and identified a subset of WHO grades 1 and 2 meningiomas with increased risk of recurrence [50]. In Table 1, a summary of correlations of WHO grading with histology, molecular alterations, methylation classes, prognosis, and location of meningiomas is presented.

## 5. Driver Signaling Mutations in Meningiomas: Potential New Targets of Therapy

Different growth factor receptors and kinases may promote the meningioma growth, including epidermal growth factor receptor (EGFR), platelet-derived growth factor receptor β (PDGFRβ), vascular endothelial growth factor receptor (VEGFR), and insulin-like growth factor receptor (IGFR) [41]. PDGFR, EGFR, and VEGFR may have a dual activity on the RAS–RAF–MEK–MAPK or FAK–PI3K–AKT pathway, resulting in growth-favoring signals in meningiomas. Moreover, after the activation of AKT, an intracellular signaling pathway acts on mTORC1 and 2 and regulates DNA replication. Other signaling pathways shown to be activated in meningiomas are the phospholipase A2–arachidonic acid–cyclooxygenase pathway [51], the phospholipase C γ1 (PLCγ1)–protein kinase C pathway (PKC) [52], and the transforming growth factor-β (TGFβ)–SMAD signaling pathway [53,54], which act as inhibitory mechanisms of meningioma growth, thus representing potential targets of treatment.

## 6. Role of Tumor Microenvironment in Meningiomas: Is It Druggable?

The meningioma microenvironment seems to play a role in tumor growth, and some data suggest that WHO grades 2 and 3 meningiomas represent a relatively immunosuppressed status. In particular, grading in meningioma negatively correlates with the amount of CD4+, CD8+, and PD-1+ lymphocytes, along with increased numbers of Treg (FOXP3+) cells in the tumor. Moreover, programmed death-ligand 1 (PD-L1) expression has been correlated with the grading of meningiomas, with PD-L1 protein detection in 40% of grade 1, 60% of grade 2, and 77–88% of grade 3 meningiomas [55]. Karimi et al. reported that PD-L1 protein expression had a patchy pattern, along with peri-vascular and peri-necrotic, membranous, and cytoplasmic immunoreactivity in both tumor and immune cells of the microenvironment [56]. Furthermore, PD-L1 expression was confined to a small subpopulation of cells (median  <  1% of cells, range 0–20% of cells), and correlated with a higher risk of early recurrence, regardless of grading, the extent of resection, and tumor diameter. Tumor-infiltrating T lymphocytes (TILs) around meningiomas may influence the prognosis. Rapp et al. analyzed the presence of TILs in 97 newly diagnosed and 62 recurrent high-grade meningiomas, reporting that a higher number of cytotoxic TILs (CD3+ CD8+ FOXP3-) were associated with an improved PFS, while recurrent meningiomas were characterized by lower numbers of TILs and proportions of PD-1+CD8+ T cells [57]. Some findings support the hypothesis that somatic genetic alterations in meningioma can potentially affect PD-L1 or other checkpoint protein expression. For instance, an increased PD-L1 expression was found in TRAF7-mutated, compared with wild-type skull-base meningiomas [58], and an increased number of CTLA4+/CD3+ lymphocytes was found in grades 2 and 3 meningiomas harboring the PI3K–AKT–mTOR pathway or SMO mutations [59]. Lastly, 40% of NF2-mutated meningiomas express PD-L1 in the surrounding microenvironment [60], suggesting that the therapeutic role of checkpoint inhibitors is worth investigating in progressive/refractory meningiomas after the failure of surgery and/or radiation therapy [61].

As M2 macrophages are the most prevalent immune cell type in meningiomas, Yeung et al. have targeted with a specific monoclonal antibody the colony-stimulating factor 1 (CSF1) and CFS1 receptor (CSF1R) expressed in myeloid cells, reporting a significant reduction in tumor growth in a murine meningioma model. This provides a strong rationale for future human clinical trials targeting the CSF1–CSF1R pathway in malignant meningiomas [62].

## 7. Systemic Therapy for Progressive/Recurrent Meningiomas: Present and Future

Patients with progressive/recurrent meningiomas, in whom surgery and/or radiation are not feasible anymore, have a limited PFS, ranging from 6-month PFS of 29% for grade 1 to 26% for grades 2 and 3 tumors [63]. To date, there is no evidence regarding the standard of care, and enrollment in clinical trials is recommended in case of disease progression [2]. A matter of debate was the choice of the best endpoint in clinical trials in surgery- and radiation-refractory meningioma: in 2019, the Response Assessment in Neuro-Oncology (RANO) group stated that an appropriate endpoint for medical therapy trials is either a 6-month PFS rate alone or in combination with radiological response [64].

Different cytotoxic chemotherapies and targeted agents have been investigated, with poor results in terms of disease control and survival, including hydroxyurea [65,66,67], temozolomide [68], irinotecan [69], trabectedin [70,71], IFN-α [72,73,74], somatostatin analogs (pasireotide [75] and octreotide [76,77]), VEGF/VEGFR inhibitors [78,79,80,81], EGFR inhibitors (erlotinib and gefitinib) [82], imatinib [83], and mifepristone [84] (Table 2).

As meningiomas are highly vascularized, anti-VEGF drugs have been largely investigated. The most employed compound was bevacizumab, which has displayed some benefit in terms of PFS (median-PFS 16.8 months, range 6.5–22 months; 6-month PFS: 73%, range 44%–93%), with particular advantage in patients with high-grade and/or multiple and/or radiation-induced meningiomas [86]. The clinical and radiological benefit of bevacizumab may derive from a pronounced inhibitory effect on tumor growth, as well as some anti-edema activity, in comparison with other targeted therapies and cytotoxic agents [87]. Given these favorable properties, bevacizumab was investigated in combination with other compounds. Shih et al. evaluated the activity of bevacizumab and the mTORC1 inhibitor everolimus; the authors reported the best radiological response for stable disease (SD) in 15 patients (88%), and 6 of these patients had SD for >12 months. Median PFS was 22 months (95% CI 4.5–26.8) and was longer for patients with grades 2 and 3 than for those with grade 1 meningiomas (22.0 months vs. 17.5 months, respectively) [79].

The Combination of Everolimus and Octreotide LAR in Aggressive Recurrent Meningiomas (CEVOREM) phase 2 trial reported a 6-month PFS of 55% (95% CI 31.3%–73.5%), and 6-month- and 12-month OS of 90% (95% CI 65.6%–97.4%) and 75% (95% CI, 50.0%–88.7%), respectively, in 20 patients with progressive meningiomas. A radiological response (decrease >50%) was achieved in 78% of patients, with a median tumor growth rate decreasing from 16.6% 3 months before inclusion to 0.02% after 3 months and 0.48% at 6 months after treatment [85].

In the era of precision medicine, we may select appropriate therapy based on specific genetic mutations. In this regard, the Alliance/NCI A071401 study initiated a genomically driven meningioma phase 2 trial in which the targeted therapy is delivered according to the mutation found in the tissue. Thus, different compounds are under investigation, including the SMO inhibitor vismodegib in *SMO*-mutant tumors, the AKT inhibitor capivasertib (AZD5363) for *AKT/PIK3CA*-mutant tumors, the CDK inhibitor abemaciclib for *NF2* or *CDK*-mutant tumors, and the FAK inhibitor GSK2256098 for *SMO/PTCH1*-mutant and *NF2*-mutant meningiomas, respectively (NCT02523014). The FAK inhibitor arm has already completed the accrual with 37 patients enrolled (12 grade 1 and 25 grades 2 and 3 meningiomas) in the trial [88]. Most patients received prior radiotherapy (75.7%) and chemotherapy (40.5%) before the start of the FAK inhibitor. One patient had a partial response, and 24 had SD as the best response to treatment. In grade 1 meningiomas, the 6-month PFS was 83% (10/12 patients; 95% CI: 52–98%). In grades 2 and 3 meningiomas, the 6–month PFS was 33% (8/24 patients; 95% CI: 16–55%). The study met the 6-month PFS endpoint both for the grade 1 and the grades 2 and 3 cohorts with excellent tolerability. However, a major concern for all targeted therapies is the resistance owing to tumor heterogeneity. Indeed, malignant meningiomas have been shown significant molecular heterogeneity within the original tumor and recurrence [89].

High-grade meningiomas may harbor an immunosuppressive microenvironment. In this regard, some studies reported that a subset of high-grade meningiomas have a high somatic mutation burden [90] that could represent a predisposing factor for a response to immune-checkpoint inhibitors [55,91]. Brastianos et al. have designed a phase 2 trial evaluating pembrolizumab in 26 patients with recurrent high-grade meningiomas (23 grade 2, and 3 grade 3 tumors). The study met the primary endpoint and achieved a 6-month PFS of 48% (90% CI 31–66), a median PFS of 7.6 months (90% CI 3.4–12.9 months), and a median OS of 20.2 months (90% CI 14.8–25.8 months). Eighteen patients had SD as the best radiological response, while no patients had complete or partial response according to RANO criteria. PD-L1 expression in pretreatment tissue was not correlated with outcome. Notably, the trial enrolled seven patients with metastatic extracranial meningiomas, of whom four achieved 6-month PFS and one patient had PFS lasting for nearly 20 months. While the trial met the primary endpoint, these results will require additional validation, and further studies are needed to identify which meningioma subtypes or tumor microenvironment patterns are correlated with the efficacy of immune-checkpoint inhibitors [92]. Conversely, a phase 2 trial on 20 patients using anti-PD1 nivolumab failed to improve 6-month PFS (42.4%, 95% CI 22.8, 60.7), although a subset of patients appeared to derive benefit (one patient obtained a partial response) [93].

Novel targeted agents are under investigation in clinical trials, including MEK inhibitor alone (selumetinib, NCT03095248) or in combination with Pi3Kα inhibitor (alpelisib, NCT03631953), VEGF inhibitor apatinib (NCT04501705), CDK-p16-Rb inhibitor ribociclib (NCT02933736), immune checkpoint inhibitors, such as nivolumab alone or in combination with ipilimumab (NCT02648997) or with stereotactic radiosurgery (NCT03604978), or sintilimab (NCT04728568) (Table 3).

## 8. Conclusions

Despite multiple studies on different cytotoxic agents performed on recurrent meningiomas in the past several years, limited evidence of efficacy and clinical benefit has been reported thus far. Hence, there is no evidence of effective systemic therapy for meningiomas. Since 2013, a genomic revolution in the biology and genomic landscape of meningiomas is underway, where the identification of molecular alterations driving the aggressiveness is translated into more reliable preclinical models that allow for rapid translation of discoveries into clinical trials. These key molecular alterations are refining the histological and molecular classification of meningiomas and allow for the stratification of patients with different outcomes and tailored treatments.

## Figures and Tables

**Table 1:** Correlations of WHO grading with histology, methylation classes, molecular alterations, location, and prognosis of meningiomas.

WHO Grading	Histology	Methylation Classes	Molecular Alterations	Location	Prognosis
Grade 1	Fibroblastic Psammomatous	MC ben-1	*NF2*	Convexity Parasagittal areas Hemispheric meninges Intraventricular Space	Good (≥95%)
	Meningothelial Secretory	MC ben-2	*TRAF7, KLF4, AKT1, SMO1, PIK3CA, POLR2A*	Skull base Basal location *Tuberculum sellae for POLR2A mutation*	Good (≥95%)
	Angiomatous Transitional Rare entities (metaplastic, microcystic, rhabdoid)	MC ben-3	Not known	Convexity Parasagittal areas Hemispheric Meninges	Good (≥95%)
Grade 2	Clear cell Chordoid Atypical	MC int-A (atypical, clear cell)	*NF2, SMARCE1, SMARCB1, SMARCA4*	Convexity Parasagittal areas Hemispheric meninges Intraventricular space *Cranial and spinal location for SMARCE1 mutation*	Intermediate (~88–90%)
		MC int-B (atypical, chordoid)	*NF2, TERT* mutations, *CDKN2A* deletion		Intermediate (~45–47%)
Grade 3	Anaplastic Rhabdoid	MC int-B	*NF2, TERT* mutations, *CDKN2A* deletion	Convexity Parasagittal areas Hemispheric meninges Intraventricular space	Intermediate (~45–47%)
		MC mal	*NF2, TERT* mutations, *CDKN2A* deletion *BAP1* (Rhabdoid)		Poor (~18–20%)

**Table 2:** Studies of systemic therapies in meningiomas.

Treatment	Type of Study	n	Results
Hydroxyurea [65]	Retrospective	60	6-month PFS: 10%
Hydroxyurea [66]	Retrospective	35	6-month PFS: 3% Median OS: 8 months
Hydroxyurea plus imatinib [67]	Phase 2	15	Early interrupted for slow accrual No significant activity
Temozolomide [68]	Phase 2	16	6-month PFS: 0% Median OS: 7.5 months
Irinotecan [69]	Phase 2	16	6-month PFS: 6% Median OS: 7 months
Trabectedin [71]	Randomized phase 2 (EORTC-1320-BTG)	90	No improvement of median PFS or median OS
Interferon-α [73]	Phase 2	35	6-month PFS: 54% Median OS: 8 months
Interferon-α [74]	Retrospective series	35	6-month PFS: 17% Median OS: 8 months
Pasireotide [75]	Phase 2	34	Grade 1: 6-month PFS: 50%: median OS: 104 weeks Grade 2–3: 6-month-PFS: 17%; median OS: 26 weeks
Octreotide [76]	Phase 2	16	6-month PFS: 44% Median OS: 7.5 months
Octreotide [77]	Phase 2	9	6-month PFS: 44% Median OS: 18.7 months
Bevacizumab [80]	Retrospective series	14	6-month PFS: 86% Median OS: not reached
Bevacizumab [81]	Retrospective series	15	6-month PFS: 44% Median OS: 15 months
Bevacizumab plus everolimus [79]	Phase 2	17	Stable disease: 88% 6-month PFS: 69% Median OS: 23.8 months
Everolimus plus octreotide [85]	Phase 2 (CEVOREM trial)	20	6-month PFS: 55% 6-month OS: 90% 12-month OS: 75% Partial response in 78% of patients
Erlotinib or gefitinib [82]	Phase 2	25	Grade 1: 6-month PFS: 25%; 12-month OS: 50% Grade 2–3: 6-month PFS: 29%; 12-month OS: 65%
Imatinib [83]	Phase 2	23	Grade 1: 6-month PFS: 45% Grade 2–3: 6-month PFS: 0%
Sunitinib [78]	Phase 2	36	6-month PFS: 42% Median PFS: 5.2 months Median OS: 24.6 months
Mifepristone [84]	Randomized phase 3 (SWOG-S9005)	164	No statistical difference between mifepristone and placebo in terms of PFS and OS

PFS: progression-free survival; OS: overall survival.

**Table 3:** Ongoing clinical trials on systemic treatments in meningiomas.

Trial ID	Type of Study	Arm of Treatment	n	Endpoints
NCT02648997	Phase 2	Nivolumab alone (Cohort 1) or in combination with ipilimumab (Cohort 2)	50	Primary: 6-month PFS Secondary: median PFS, median OS, ORR, safety
NCT03631953	Phase 1	Alpelisib in combination with trametinib	25	Primary: DLT
NCT04728568	Prospective	Sintilimab	15	Primary: PFS Secondary: OS
NCT04501705	Prospective	Apatinib	29	Primary: 6-month PFS Secondary: ORR, OS
NCT03604978	Phase 1–2	Nivolumab alone or plus ipilimumab in combination with fractionated SRS	15	Primary: DLT, safety, ORR Secondary: median PFS, median OS, changes in peripheral T-cells
NCT02933736	Early phase 1	Ribociclib	48	Primary: plasma exposure, CSF penetration, brain accumulation of ribociclib
NCT02523014	Phase 2	Vismodegib or FAK inhibitor, or GSK2256098 or capivasertib, or abemaciclib based on molecular screening	124	Primary: 6-month PFS, ORR Secondary: median PFS, median OS, safety
NCT04659811	Phase 2	Pembrolizumab plus SRS	90	Primary: 12-month PFS Secondary: median PFS, median OS
NCT04374305	Phase 2	Brigatinib	80	Primary: radiological response rate Secondary: safety
NCT03095248	Phase 2	Selumetinib	34	Primary: change in hearing response, response rate of other NF2-related tumors (including meningiomas)
NCT04541082	Phase 1	ONC206	102	Primary: MTD

PFS: progression-free survival; OS: overall survival; ORR: objective response rate; DLT: dose-limiting toxicity; SRS: stereotactic radiosurgery; MTD: maximum tolerated dose.
